# miR-486-5p Restrains Extracellular Matrix Production and Oxidative Damage in Human Trabecular Meshwork Cells by Targeting TGF-*β*/SMAD2 Pathway

**DOI:** 10.1155/2022/3584192

**Published:** 2022-02-23

**Authors:** Le Xu, Yiming Zhang, Hua Long, Bo Zhou, Haibo Jiang

**Affiliations:** ^1^Department of Ophthalmology, Suizhou Hospital, Hubei University of Medicine, Suizhou 441300, Hubei, China; ^2^Department of Orthopedics, Suizhou Hospital, Hubei University of Medicine, Suizhou 441300, Hubei, China

## Abstract

**Background:**

Glaucoma is characterized by elevated intraocular pressure caused by aqueous outflow dysfunction. Trabecular meshwork plays a key role in controlling intraocular pressure by modulating aqueous outflow. This study investigated the protective effects of miR-486-5p in H_2_O_2_-stimulated human trabecular meshwork cells (TMCs).

**Methods:**

TMCs were disposed with 300 *μ*M H_2_O_2_ to establish oxidative damage models *in vitro*. miR-486-5p mimics and its controls were transfected into TMCs, and cell apoptosis and extracellular matrix production (ECM) genes were measured by flow cytometry, western blotting, and immunofluorescence staining. Activities of superoxide dismutase (SOD) and malondialdehyde (MDA) were also assayed. Online tools and luciferase reporter assays were used to uncover the relationship between miR-486-5p and the TGF-*β*/SMAD2 pathway.

**Results:**

We found that H_2_O_2_-induced oxidative damage in TMCs and miR-486-5p was downregulated in H_2_O_2_-stimulated TMCs. Overexpression of miR-486-5p mitigated H_2_O_2_-induced oxidative damage by inhibiting apoptosis, reducing cleaved caspase-3/9 expression, reducing MDA levels, and increasing SOD levels as well as downregulating ECM genes. SMAD2 was demonstrated to be targeted by miR-486-5p, and miR-486-5p inhibited TGF-*β*/SMAD2 signaling in H_2_O_2_-stimulated TMCs. Additionally, SMAD2 was upregulated by H_2_O_2_, and SMAD2 upregulation abrogated the protective effects of miR-486-5p against H_2_O_2_-induced injury.

**Conclusion:**

miR-486-5p restrains H_2_O_2_-induced oxidative damage in TMCs by targeting the TGF-*β*/SMAD2 pathway.

## 1. Introduction

Glaucoma is a complex neurodegenerative disorder characterized by progressive optic neuropathy, which can result in blurred vision and even blindness [1]. The main risk factors of glaucoma include elevated intraocular pressure and insufficient blood supply to the optic nerve [2]. Currently, lowering intraocular pressure is the mainstream treatment in retarding the process of glaucomatous neuropathy, which can be achieved by medication, conventional surgery, and laser [3]. Although these remedies may rescue remaining eyesight, many patients fail to reach intraocular pressure targets [4]. Elevated intraocular pressure is a direct consequence of increased resistance to aqueous humor outflow [5]. It has been recognized that the trabecular meshwork (TM) exerts the main function on aqueous humor circulation [6, 7]. Studies show that an extraordinary increase of extracellular matrix (ECM) molecules such as collagen and fibronectin in the TM is the main cause of outflow resistance [8, 9]. Therefore, there is an unmet need to elucidate the pathological mechanism related to excessive production of ECM from glaucomatous TM.

The transforming growth factor-*β* (TGF-*β*) family modulates a range of cellular processes, including ECM synthesis, cell proliferation, and differentiation [10, 11]. It is also recognized as a crucial profibrotic mediator in fibrotic disease. Studies show that the TGF-*β*/SMAD signaling pathway exerts key effects on regulating ECM synthesis in fibrotic processes [12]. In glaucomatous optic neuropathy, the TGF-*β*/SMAD pathway regulates key ECM genes, such as collagen, laminin, and fibronectin in the TM, which is associated with elevated intraocular pressure [13–15]. Previous reports indicated that TGF-*β*2-subjected human TMCs elicits a significant increase in SMAD2/3 signaling as well as an increase in collagen protein content [16]. Downregulation of SMAD7 interrupts the effects of TGF-*β*2 on the expression of several ECM components, and SMAD7 is a key molecular switch to inhibit TGF-*β*2 signaling in the TM [17, 18]. Additionally, nonpigmented ciliary epithelium-derived extracellular vesicles loaded with SMAD7 siRNA attenuate Wnt signaling in TM cells, which may be beneficial as a therapeutic target to lower intraocular pressure [19]. Therefore, it is needed to discover potential targets in the TGF-*β*/SMAD pathway to restrain ECM production in the TM.

MicroRNAs (miRNAs) contain approximately 18–22 nucleotides and participate in the regulation of the biological process by controlling gene expression post-transcriptionally [20]. In glaucoma, differentially expressed miRNAs in the aqueous humor and blood of patients have been identified and compared to matched control individuals [21, 22]. Increasing studies have focused on the roles of miRNAs in modulating cellular function of the TM under various pathological conditions. Zhang et al. demonstrated that miR-181a improves the survival of TMCs under oxidative stress by blocking NF-*κ*B signaling [23]. Overexpression of miR-183 reinforces DNA damage and promotes cellular senescence in the TM after ultraviolet irradiation by targeting KIAA0101 [24]. Additionally, miRNAs were proven to exert key functions in maintaining ECM homeostasis [25, 26]. For example, miR-137 facilitates cell proliferation and downregulates ECM protein expression levels in H_2_O_2_-stimulated human TMCs through the YAP/TAZ pathway [27]. miR-483-3p attenuates ECM production in TMCs by targeting SMAD4 [28].

Small RNA sequencing results showed that miR-486-5p was downregulated in the aqueous humor of glaucoma patients [29]. Moreover, miR-486-5p was shown to inhibit TGF-*β*2-mediated ECM remodeling via inhibition of SMAD2 [30]. Thus, a future study is needed to explore whether restoration of miR-486-5p expression could block SMAD2 signaling in the TMCs, blocking ECM remodeling under mechanical stress.

## 2. Materials and Methods

### 2.1. Cell Culture

Human TMCs were obtained from ScienCell Research Laboratories (cat. no. #6590; San Diego, CA, USA). Cells were cultured at 37 °C with 5% CO_2_ in DMEM with L-glutamine, 10% FBS, 110 mg/mL sodium pyruvate, 100 units/mL penicillin, 100 *μ*g/mL streptomycin sulfate, 100 *μ*M nonessential amino acids, and 0.25 *μ*g/mL amphotericin B (all obtained from Gibco, Carlsbad, CA, USA). Cells in passages 3–5 were used for experiments. To establish oxidative stress models, TMCs were treated with 100, 200, 300, and 400 *μ*M H_2_O_2_ (Gibco) at 37°C for 12 h, and nontreated cells served as a control. Cell viability was measured to evaluate the injury induced by H_2_O_2_ in TMCs, and 300 *μ*M H_2_O_2_ was used for further assays.

### 2.2. Cell Transfection

TMCs (1 × 10^6^ cells/mL) were seeded in 6-well plates for 24 h and were subsequently transfected with miR-486-5p mimics (50 nM; UCCUGUACUGAGCUGCCCCGAG; RiboBio, Guangzhou, China), negative control (NC) mimics (50 nM; UAUCCGGCCUGCGCCGUUAGCA; RiboBio), SMAD2 short hairpin RNA (sh-SMAD2; 50 nmol/L; GCAGAACTATCTCCTACTACTTTCAAGAGAAGTAGTAGGAGATAGTTCTGCTTTTTT; GenePharma, Shanghai, China), and sh-NC (50 nmol/L; TTATCAACAAGGTCCTCCTACTTCAAGAGAGTAGGAGGACCTTGTTGATAATTTTTT; GenePharma) using Lipofectamine 2000 (cat. no. 11668–027; Invitrogen, CA, USA) following the guidelines by manufacturer's protocol. The full length of SMAD2 was synthesized and subcloned into the pcDNA3.1 (cat. no. HG-VPI0001; Invitrogen; Thermo Fisher Scientific, Inc.) plasmid to produce pcDNA3.1/SMAD2, and an empty pcDNA3.1 vector acted as the negative control. After 48 h post-transfection, RT-qPCR was used to detect transfection efficiency. TMCs were subjected to oxidative stress with 300 *μ*M H_2_O_2_ at 37°C for 12 h and collected for further use.

### 2.3. Flow Cytometry for Transfection Efficiency Detection

The transfected cells were collected and treated with 0.25% trypsin followed by resuspension in PBS. Cells were prepared into single-cell suspension which was then centrifuged at 800 rpm for 5 min. After that, the supernatant was removed, and cells were washed with PBS. Cells (1 × 10^6^ cells/mL) were then subjected to transfection efficiency detection by a FACScan flow cytometer (Becton–Dickinson, CA, USA).

### 2.4. RT-qPCR

Following treatment, total RNA was extracted from TMCs using TRIzol reagents (Invitrogen) and mirPremier microRNA isolation kits (Sigma-Aldrich, MO, USA) as per the manufacturer's protocols. The RNA extracts were reverse-transcribed into cDNA using M-MLV reverse transcriptase (cat. no. M1701; Promega, MI, USA) and miRNA First-Strand cDNA synthesis kits (cat. no. QP114; GeneCopoeia, Rockville, MD, USA), respectively. The qPCR was performed on the ViiA^TM^ 7 real-time PCR system (ABI, CA, USA) using FastStart Universal SYBR Green Master (cat. no. 4913850001; Roche, Switzerland). Relative expression was quantified normalization to GAPDH and U6. The primers used for qPCR are listed: miR-486-5p 5′-CTCGCTTCGGCAGCACA-3′ (forward) 5′-ACGCTTCACGAATTTGCGT-3′ (reverse); SMAD2 5′-CAATCGCCCATTCCCCTCTT-3′ (forward) 5′-AGTCTCTTCACAACTGGCGG-3′ (reverse); U6 5′-CTCGCTTCGGCAGCACA-3′ (forward) 5′-AACGCTTCACGAATTTGCGT-3′ (reverse); GAPDH 5′-ACTGAGCAAGAGAGGCCCTA-3′ (forward) 5′-TATGGGGGTCTGGGATGGAA-3′ (reverse).

### 2.5. Western Blotting

After washing twice with cold PBS, TMCs were lysed in RIPA buffer (cat. no. E-BC-R327; Elabscience, Wuhan, China) to extract total protein. The protein samples (30 *μ*g) were denatured and separated by 10% SDS/PAGE. Protein was then moved onto PVDF membranes and blocked with 5% skimmed milk containing PBS to block nonspecific binding. Subsequently, the membranes were cultured overnight at 4°C with primary detection antibodies, including cleaved caspase-3 (ab32042; Abcam, Cambridge, USA), cleaved caspase-9 (# 20750S, Cell Signaling Technology, MA, USA), collagen I (ab138492), fibronectin (ab268020), laminin (ab108536), SMAD2 (ab40855), TGF-*β* (ab215715), and *β*-actin (# 4970S; Cell Signaling Technology). Appropriate secondary antibodies were then incubated with the blots for 1 h at room temperature. After washing, the signals were monitored with ECL Advance reagents (GE Healthcare, Braunschweig, Germany) and analyzed with Image Lab v6.0 software.

### 2.6. MTT Assay

After treatment with concentrations of H_2_O_2_, TMCs were seeded in 96-well plates at 3 × 10^3^ cells/well in a 37°C incubator. Next, 20 *μ*L MTT solution (5 mg/mL; Sigma-Aldrich) was added and incubated for 4 h at 37°C. The medium was removed, and the residue was dissolved with 100 *μ*L dimethyl sulfoxide for 15 min. The optical density at 490 nm was measured with a SpectraMax M2e microplate reader (Molecular Devices, CA, USA).

### 2.7. Flow Cytometry

Annexin V-FITC/PI Apoptosis Kits (cat. no. 70-AP101-100; Hangzhou, China) was utilized to analyze apoptosis. After treatment, TMCs were washed with cold PBS and suspended in 1× binding buffer (Sigma-Aldrich). Subsequently, 500 *μ*L cell suspension (1 × 10^6^ cell/mL) was added to a flow tube. Cells were then stained with 5 *μ*L Annexin V-FITC and 10 *μ*L PI, respectively, for 15 min in the dark. Finally, the stained cells were analyzed with a FACScan flow cytometer (Becton–Dickinson, CA, USA) and FlowJo software version 7.6.1.

### 2.8. Immunofluorescence Staining

Briefly, TMCs were fixed with 4% paraformaldehyde for 30 min, washed with PBS, and permeabilized with 0.3% Triton X-100 for 5–10 min. Subsequently, cells were blocked in 5% bovine serum albumin containing PBS for 30 min at room temperature. Next, cells were cultured overnight at 4°C with primary detection antibodies, including collagen I (cat. no. 67288-1-lg; Proteintech, Wuhan, China), fibronectin (cat. no. 66042-1-lg; Proteintech), and laminin (23498-1-AP). After washing with PBS, Alexa Fluor 555-conjugated rabbit IgG (Abcam) was added and incubated with cells for 30 min at room temperature. DAPI (Sigma-Aldrich) was then used for DNA counterstaining. Fluorescent cells were observed with a confocal laser scanning microscope (LSM710; Carl Zeiss, Germany).

### 2.9. ELISA

The levels of superoxide dismutase (SOD) and malondialdehyde (MDA) in the cell medium were analyzed with SOD assay kits (cat. no. ab65354) and MDA assay kits (cat. no. ab118970) as per the manufacturer's protocols.

### 2.10. Bioinformatics Analysis

TargetScan (http://www.targetscan.org/vert_70/), starBase (https://starbase.sysu.edu.cn/), miRDB (http://mirdb.org/), and TargetRank (http://hollywood.mit.edu/targetrank/) were used to predict the targets of miR-486-5p.

### 2.11. Luciferase Reporter Assay

HEK293T cells or TMCs were seeded in 24-well plates. Luciferase reporter plasmids were constructed: pmirGLO-SMAD2-Wt containing the SMAD2 3'-UTR in the binding site for miR-486-5p and pmirGLO-SMAD2-Mut with a mutation at the predicted miR-486-5p binding site. After being cultured to 80% confluency, cells were transfected with 800 ng SMAD2-Wt or SMAD2-Mut plasmids, together with 20 nmol miR-486-5p mimics or NC mimics. After 48 h post-transfection, the activities of Renilla luciferase that were normalized to firefly luciferase activities were measured using a dual-luciferase reporter system (Promega).

### 2.12. Statistical Analysis

All tests were conducted in at least 3 biological replicates. Comparisons were performed using unpaired Student's *t*-test and one-way ANOVA. Data were processed with SPSS v. 23.0 (IBM Corp., NY, USA) and are expressed as the mean ± SD. Statistical significance was set at *p* < 0.05.

## 3. Results

### 3.1. miR-486-5p Was Downregulated in H_2_O_2_-Stimulated TMCs

**Figure 1:**
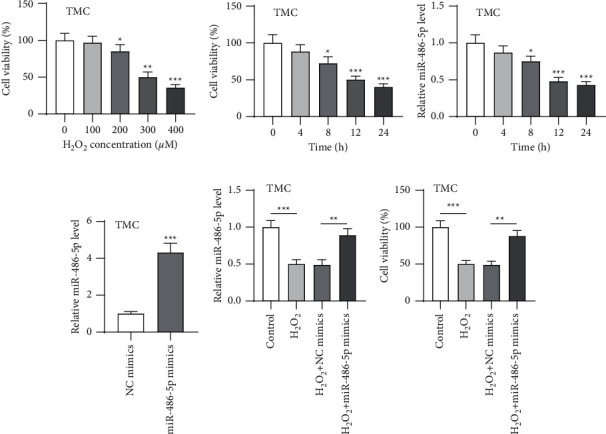
miR-486-5p was downregulated in H_2_O_2_-stimulated TMCs. (a) After TMCs were treated for 12 h with varying concentrations of H_2_O_2_ (0–400 *μ*M), viability was detected by MTT. After TMCs were treated with 300 *μ*M H_2_O_2_ for 0–24 h (b) viability was detected by MTT. (c) The miR-486-5p level was assessed by RT-qPCR. Transfected and nontransfected cells were stimulated with 300 *μ*M H_2_O_2_ for 12 h and nontreated cells acted as the control. (d, e) Overexpression efficiency of miR-486-5p mimics in nontreated cells and H_2_O_2_-treated TMCs was evaluated by RT-qPCR. (f) Viability was detected by MTT. ^*∗*^*p* < 0.05; ^∗∗^*p* < 0.01; ^∗∗∗^*p* < 0.001.

We established the oxidative injury model in TMCs using H_2_O_2_. TMCs were subjected to varying concentrations of H_2_O_2_ (0–400 *μ*M) for 12 h, followed by measurement for cell viability by the MTT assay. We observed that the viability was suppressed by H_2_O_2_ at 100 *μ*M, 200 *μ*M, 300 *μ*M, and 400 *μ*M (all *p* < 0.05) (Figure 1(a)). We selected 300 *μ*M H_2_O_2_ to treat TMCs for further experiments since the IC50 of viability was shown at 300 *μ*M. The MTT results showed that 300 *μ*M H_2_O_2_ greatly repressed the viability of TMCs in a time-dependent manner, and the IC50 was presented at 12 h (Figure 1(b)). Next, the miR-486-5p level in H_2_O_2_-stimulated TMCs was determined by RT-qPCR, which indicated that H_2_O_2_ time-dependently downregulated miR-486-5p expression (Figure 1(c)), implying that miR-486-5p may relate to the H_2_O_2_-induced injury in TMCs. The miR-486-5p mimics were transfected into TMCs to upregulate miR-486-5p. RT-qPCR demonstrated that miR-486-5p was successfully upregulated following transfection (Figure 1(d)). Next, transfected and nontransfected cells were stimulated with 300 *μ*M H_2_O_2_ for 12 h, and nontreated cells acted as the control. We also verified that miR-486-5p mimics upregulated miR-486-5p expression in H_2_O_2_-treated TMCs (Figure 1(e)). The MTT assay showed that miR-486-5p overexpression significantly elevated the viability of H_2_O_2_-treated cells (Figure 1(f)).

### 3.2. miR-486-5p Overexpression Mitigated H_2_O_2_-Induced Injury in TMCs

**Figure 2:**
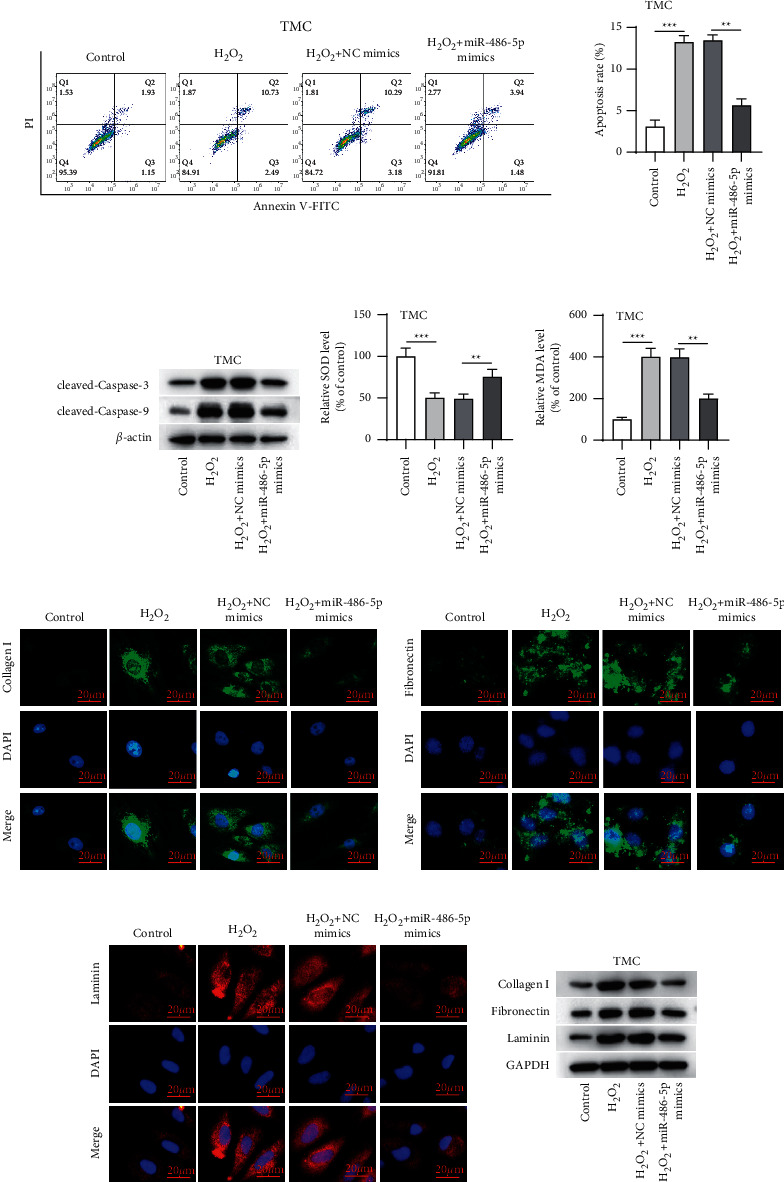
miR-486-5p overexpression mitigated H_2_O_2_-induced injury in TMCs. After TMCs were disposed with 300 *μ*M H_2_O_2_ alone, 300 *μ*M H_2_O_2_ plus NC mimics alone, or 300 *μ*M H_2_O_2_ plus miR-486-5p mimics, (a) apoptosis was assessed by flow cytometry; (b) protein expression of cleaved caspase-3/9 was determined by western blotting; (c, d) the SOD and MDA levels were assayed; (e, f) content of collagen I, fibronectin, and laminin was tested by the immunofluorescence staining assay; and (g) expression of collagen I, fibronectin, and laminin was tested by western blotting. ^∗∗^*p* < 0.01; ^∗∗∗^*p* < 0.001.

To determine the role of miR-486-5p, TMCs overexpressing miR-486-5p were exposed to H_2_O_2_. Under oxidative stress, the apoptosis of TMCs was significantly elevated compared to the control condition; however, miR-486-5p overexpression led to a marked inhibition in cell apoptosis (Figure 2(a)). Western blotting also showed that miR-486-5p overexpression reduced the promotive effects of H_2_O_2_ on the expression levels of cleaved caspase-3/9 in TMCs (Figure 2(b)). SOD is the main antioxidative metalloenzyme that is able to resist oxygen-free radicals; MDA is the final product of peroxidation [31]. We thus assessed the levels of SOD and MDA in TMCs under the treatment of H_2_O_2_. As presented in Figures 2(c) and 2(d), H_2_O_2_ notably increased the SOD and MDA levels compared to the control, but these changes were reversed by miR-486-5p overexpression. We then evaluated the effects of miR-486-5p on ECM in H_2_O_2_-stimulated TMCs using the immunofluorescence staining assay and discovered that miR-486-5p overexpression could significantly downregulate ECM genes, including collagen I, fibronectin, and laminin expression (Figures 2(e)–2(g)). Western blotting further indicated the same effects of miR-486-5p overexpression on the expression levels of collagen I, fibronectin, and laminin (Figure 2(h)).

### 3.3. miR-486-5p Targeted SMAD2

**Figure 3:**
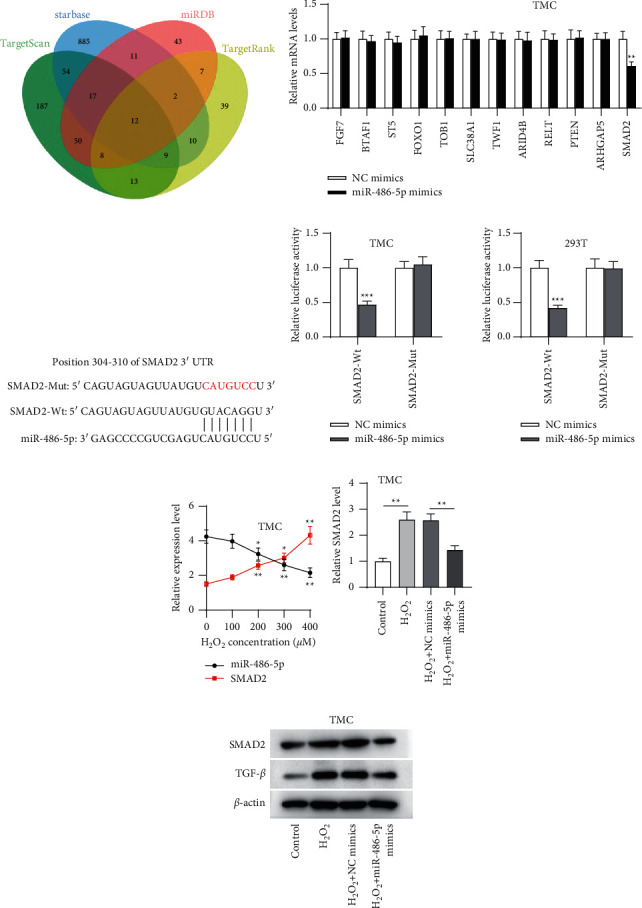
miR-486-5p targets SMAD2. (a) Twelve genes (FGF7, BTAF1, ST5, FOXO1, TOB1, SLC38A1, TWF1, ARID4B, RELT, PTEN, ARHGAP5, and SMAD2) that were overlapped among target-predicting algorithms are shown as a Venn diagram. (b) Expression of candidate targets in miR-486-5p-overexpressing TMCs was assessed by RT-qPCR. (c) Analysis of the SMAD2 3'-UTR showed a binding site for miR-486-5p. (d) In miR-486-5p-overexpressing TMCs and HEK293T cells, the luciferase activities of reporters with a wild type or mutant SMAD2 3'-UTR were detected. (e) Relative expression of miR-486-5p and SMAD2 in TMCs treated with varying concentrations of H_2_O_2_ for 12 h are quantified by qPCR. After TMCs were disposed with 300 *μ*M H_2_O_2_ alone, 300 *μ*M H_2_O_2_ plus NC mimics alone, or 300 *μ*M H_2_O_2_ plus miR-486-5p mimics, (f) the SMAD2 level was determined by RT-qPCR; and (g) protein expression of SMAD2 and TGF-*β* was determined by western blotting. ^∗∗^*p* < 0.01; ^∗∗∗^*p* < 0.001.

To identify the targets of miR-486-5p, 4 target-predicting algorithms (TargetScan, starBase, miRDB, and TargetRank) were used. Twelve genes (FGF7, BTAF1, ST5, FOXO1, TOB1, SLC38A1, TWF1, ARID4B, RELT, PTEN, ARHGAP5, and SMAD2) that were overlapped among these algorithms are shown as a Venn diagram (Figure 3(a)). Of the 12 candidate genes, SMAD2 was revealed to be downregulated in miR-486-5p-overexpressing TMCs, while the other genes had no apparent response (Figure 3(b)). Analysis of the SMAD2 3'-UTR showed a binding site for miR-486-5p (Figure 3(c)). Next, the mutations in the binding site were generated to abolish the miR-486-5p-SMAD2 3'-UTR interaction (Figure 3(c)). In miR-486-5p-overexpressing TMCs and HEK293T cells, the luciferase activities of reporters with a wild type SMAD2 3'-UTR were markedly suppressed, whereas the reporters with the mutant binding site in the SMAD2 3'-UTR had no changes (Figure 3(d)). This suggested that miR-486-5p directly targets SMAD2 3'-UTR. In addition, H_2_O_2_ concentration-dependently downregulated the miR-486-5p level, and SMAD2 showed an opposite trend in the expression level (Figure 3(e)). We further demonstrated that miR-486-5p overexpression negatively regulated the SMAD2 level in TMCs in the presence of H_2_O_2_, as RT-qPCR showed (Figure 3(f)). Additionally, the protein levels of SMAD2 and TGF-*β* were increased after H_2_O_2_ treatment and were reduced after miR-486-5p overexpression (Figure 3(g)), suggesting that miR-486-5p inhibits TGF-*β*/SMAD2 signaling in H_2_O_2_-stimulated TMCs.

### 3.4. SMAD2 Downregulation Attenuated H_2_O_2_-Induced Injury in TMCs

**Figure 4:**
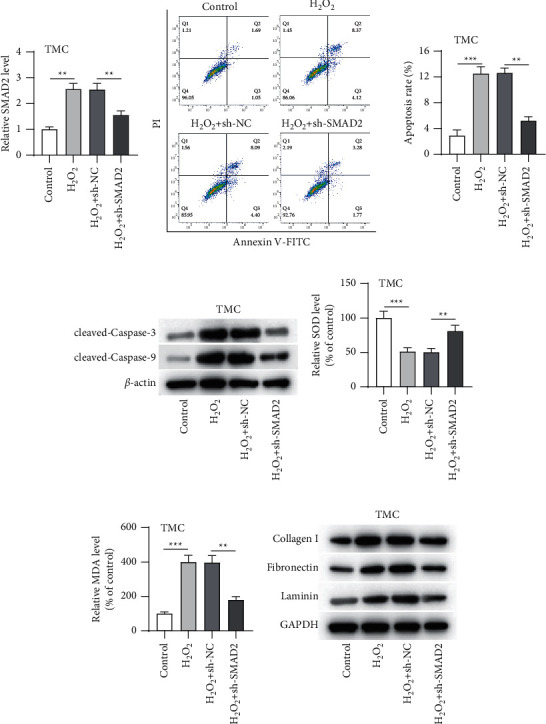
SMAD2 downregulation attenuated H_2_O_2_-induced injury in TMCs. After TMCs were disposed with 300 *μ*M H_2_O_2_ alone, 300 *μ*M H_2_O_2_ plus sh-NC alone, or 300 *μ*M H_2_O_2_ plus sh-SMAD2, (a) the SMAD2 level was determined by RT-qPCR; (b) apoptosis was assessed by flow cytometry; (c) protein expression of cleaved caspase-3/9 was determined by western blotting; (d, e) the SOD and MDA levels were assayed; (f) expression of collagen I, fibronectin, and laminin was tested by western blotting. ^∗∗^*p* < 0.01; ^∗∗∗^*p* < 0.001.

Next, we explored the effects of SMAD2 on H_2_O_2_-induced injury in TMCs. The sh-SMAD was transfected into TMCs to downregulate SMAD2. RT-qPCR showed that SMAD2 was knocked down by sh-SMAD2 compared to the control (sh-NC) (Figure 4(a)). Downregulated SMAD2 markedly decreased the apoptosis that was elevated by H_2_O_2_ (Figure 4(b)). Western blotting showed reduced protein expression of cleaved caspase-3/9 in H_2_O_2_-treated TMCs after SMAD2 downregulation (Figure 4(c)). Moreover, SMAD2 downregulation abrogated the effects of H_2_O_2_ on the SOD and MDA levels in TMCs (Figures 4(d) and 4(e)). ECM genes, including collagen I, fibronectin, and laminin expression, were significantly upregulated by H_2_O_2_, but this effect was abolished by SMAD2 downregulation (Figure 4(f)).

### 3.5. miR-486-5p Regulated H_2_O_2_-Induced Injury in TMCs by Targeting SMAD2

**Figure 5:**
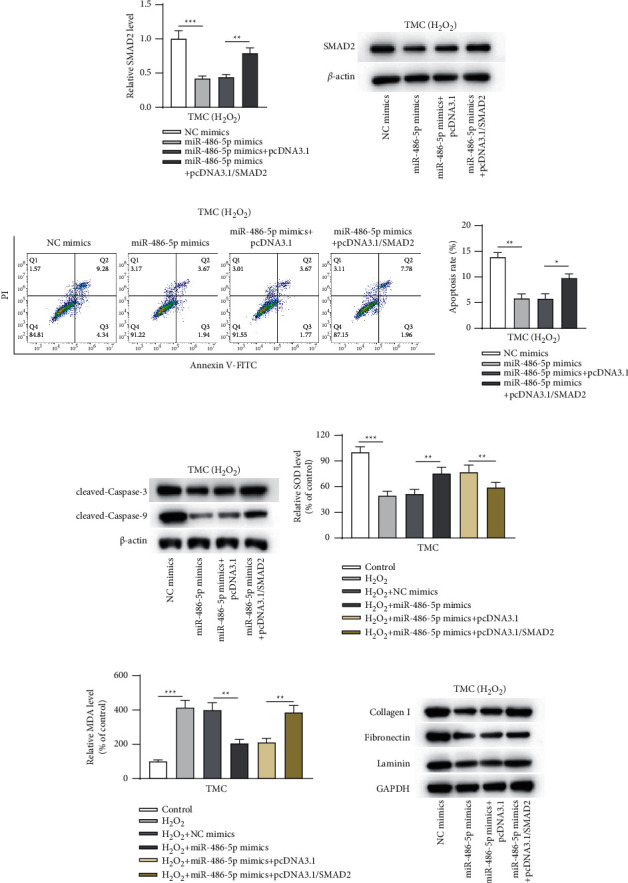
miR-486-5p regulated H_2_O_2_-induced injury in TMCs by targeting SMAD2. After H_2_O_2_-treated TMCs were transfected with NC mimics alone, miR-486-5p mimics alone, miR-486-5p mimics plus pcDNA3.1 alone, or miR-486-5p mimics plus pcDNA3.1/SMAD2, (a, b) mRNA and protein expression of SMAD2 was assessed by RT-qPCR and western blotting, respectively; (c) apoptosis was assessed by flow cytometry; (d) protein expression of cleaved caspase-3/9 was determined by western blotting; (e, f) the SOD and MDA levels were assayed; and (g) expression of collagen I, fibronectin, and laminin was tested by western blotting. ^*∗*^*p* < 0.05; ^∗∗^*p* < 0.01; ^∗∗∗^*p* < 0.001.

Rescue assays were conducted to assess the role of the miR-486-5p/SMAD2 axis in H_2_O_2_-induced injury in TMCs. As shown, transfection of miR-486-5p mimics + pcDNA3.1/SMAD2 reversed the decrease in SMAD2 mRNA and protein expression mediated by miR-486-5p mimics (Figure 5(a) and 5(b)). Furthermore, the inhibited apoptosis caused by miR-486-5p mimics was restored after transfection with miR-486-5p mimics + pcDNA3.1/SMAD2 (Figure 5(c)). The changes in protein expression of cleaved caspase-3/9 also demonstrated this (Figure 5(d)). The miR-486-5p overexpression-induced effects on the SOD and MDA levels were reversed by SMAD2 overexpression (Figures 5(e) and 5(f)). As revealed in Figure 5(g), SMAD2 overexpression restored the collagen I, fibronectin, and laminin expression decreased by miR-486-5p overexpression.

## 4. Discussion

The TGF-*β*/SMAD signaling pathway is required for fibrotic progress and ECM production, frequently detected in types of pathological conditions or diseases. For example, idiopathic pulmonary fibrosis has the characteristics of excessive fibroblast proliferation and excessive deposition of ECM components [32, 33]. It was proven that protein tyrosine phosphatase *α* induces profibrotic signaling pathways via controlling cellular responses to TGF-*β* [34]. Emerging research has revealed that TGF-*β* is able to upregulate collagen-1 and fibronectin expression [35, 36]. The SMAD system is the predominant intracellular signaling of TGF-*β*. In addition, other pathways, such as p38 MAPK and PI3K/Akt, can respond to TGF-*β* and promote ECM synthesis via SMAD pathways [36, 37].

TGF-*β*/SMAD signaling also plays a role in the TM, which is commonly overactive in glaucomatous neuropathy [9, 38]. Both non-SMAD and SMAD pathways are involved in TGF-*β*-induced lysyl oxidase synthesis, which takes major responsibility for elevated intraocular pressure [39]. As the members of the SMAD system, SMAD2/3 are shown to be responsible for TGF-*β*2-induced ECM deposition in the TM [40]. Recent studies that have focused on the roles of miRNAs in modulating the biological functions of the TM contribute to identifying more biomarkers for the treatment of glaucoma [27, 41]. It was demonstrated by Hubens *et al.* that hsa-miR-486-5p has a downregulated level in glaucoma patients [29]. In the lens, miR-486-5p targets SMAD2 to repress TGF-*β*2-induced ECM remodeling [30]. Additionally, miR-486-5p suppresses non-small cell lung cancer by targeting the TGF-*β*/SMAD2 signaling [42]. If miR-486-5p could target SMAD2 in TMCs, elevated ECM remodeling may be detected in glaucoma patients. This also may contribute to elevated production of fibronectin that is related to reduced aqueous humor outflow and increased stiffness [43]. Therefore, investigating whether restoring miR-486-5p level can downregulate TGF-*β*/SMAD2 signaling in TMCs under oxidative stress, preventing ECM remodeling and cell death, is necessary. In this research, we demonstrated a targeted relationship between SMAD2 and miR-486-5p in TMCs under oxidative stress. miR-486-5p was downregulated and SMAD2 was upregulated by H_2_O_2_. Functionally, miR-486-5p upregulation inhibited apoptosis, reduced the MDA level, and increased the SOD level as well as downregulated ECM genes, showing the same function as SMAD2 knockdown. Moreover, SMAD2 upregulation abrogated the protective effects of miR-486-5p against H_2_O_2_-induced injury.

Overall, oxidative stress induces marked alternations in miRNA expression levels, which contributes to some cellular responsiveness to oxidative stress in TMCs. Concretely speaking, we demonstrated that miR-486-5p restrains oxidative stress-induced ECM synthesis via targeting SMAD in TMCs. Therefore, the miR-486-5p/SMAD2 axis may act as a regulatory mechanism limiting ECM accumulation induced by oxidative stress in the aqueous humor outflow pathway. In future work, we would conduct animal experiments to persuade our findings.

## Data Availability

The datasets used or analyzed during the current study are available from the corresponding author on reasonable request.
